# Public Perspectives on Palliative and Hospice Care: Social Media Content Analysis Using Topic Modeling and Multiclass Sentiment Analysis

**DOI:** 10.2196/70836

**Published:** 2025-09-16

**Authors:** Aeri Kim, Kyungmi Woo

**Affiliations:** 1 The Research Institute of Nursing Science Seoul National University Seoul Republic of Korea; 2 College of Nursing Seoul National University Seoul Republic of Korea; 3 Center for World-leading Human-care Nurse Leaders for the Future by Brain Korea 21 (BK 21) four project College of Nursing Seoul National University Seoul Republic of Korea

**Keywords:** palliative care, hospice, social media, Naver, natural language processing, topic modeling, sentiment analysis

## Abstract

**Background:**

Palliative care enhances dignity and quality of life for patients with serious illnesses by managing distressing symptoms and supporting families. However, inadequate awareness and misconceptions often hinder patients and their families from accessing these services. Understanding of public perspectives on palliative and hospice care can facilitate the development of targeted educational resources and awareness campaigns. As social media becomes an important source of health information, analyzing such publicly available web-based sources can yield valuable insights into perceptions of palliative and hospice care.

**Objective:**

This study analyzed public perspectives posted on a popular social media platform in South Korea to understand perceptions, challenges, needs, and sentiments related to palliative and hospice care.

**Methods:**

Data were collected from Naver Knowledge iN, a popular web-based public forum in South Korea, encompassing 34,501 texts posted between 2002 and 2024. After applying inclusion and exclusion criteria, 9072 relevant perspectives were analyzed. Contextualized topic modeling was used to identify themes, and the optimal model was selected based on coherence, diversity scores, and expert feedback. In addition, multiclass sentiment analysis using a fine-tuned Korean bidirectional encoder representations from transformers (KoBERT) model classified sentiments into 6 categories. The multiclass sentiment model’s performance was evaluated using accuracy, precision, recall, and *F*_1_-score.

**Results:**

Social media discussions on palliative and hospice care have increased steadily over time. Topic modeling identified 9 themes, with “ethical and legal concerns” and “medical care in hospitals” peaking in recent years, suggesting growing public interest in these areas. “Emotional and psychological support” emerged as the predominant theme, reflecting a significant need for psychosocial assistance among patients and their families. Sentiment analysis revealed that “sadness,” “anxiety,” and “neutral” were common emotions across many topics. Notably, themes such as “emotional and psychological support,” “disease treatment outcomes and prognosis,” “medical care in hospitals,” “financial issues,” and “symptom management” were predominantly associated with “sadness,” while “administrative and volunteer services,” “ethical and legal concerns,” and “nutrition management” were more closely linked with “anxiety.”

**Conclusions:**

This study highlights public concerns about palliative and hospice care in South Korea, including ethical dilemmas, caregiving burden, and emotional distress. Findings underscore the need for communication strategies that address informational, emotional, and psychological needs. Culturally sensitive, interactive communication tools, such as artificial intelligence–powered chatbots and public education campaigns, may help dispel misconceptions and promote timely, informed decisions about palliative and hospice care.

## Introduction

Palliative care manages pain and other distressing symptoms in patients with serious diseases, ensuring dignity and quality of life throughout the treatment process while providing support to patients’ families [1]. Palliative care offers physical, psychological, social, and spiritual support from a multidisciplinary team that includes doctors, nurses, social workers, and therapists. As the global population continues to age and the concept of “dying in place,” which refers to patients choosing to die in their usual place of residence, gains broader recognition, the role of palliative care in community settings becomes increasingly important, especially in shaping public perceptions and responding to temporal shifts in care needs [2]. A concept analysis conducted in the United States defined key precursors and outcomes of palliative care. The key precursors to its initiation include severe, progressive, or terminal diseases. Palliative care outcomes enhance patients’ dignity and quality of life, promote the development of advanced care plans, and reduce psychological stress experienced by families, thereby supporting them in the bereavement process [3].

In South Korea, palliative care was formalized in 2011 with hospice services primarily for patients with terminal cancer, followed by the 2016 Act on Decisions on Life-Sustaining Treatment, which expanded services to include other serious illnesses. Services are provided through Regional Hospice Centers and institutions specialized in hospice care, offering inpatient, home-based, consultative care, and pediatric palliative care. In 2024, South Korea’s Ministry of Health and Welfare announced the “Second Comprehensive Plan for Hospice and Life-Sustaining Treatment (2024-2028)” to ensure a dignified end-of-life experience for everyone. This comprehensive plan aims to enhance policy accessibility, expand end-of-life care infrastructure, strengthen the competencies of service providers and staff, raise public awareness and policy recognition, and reinforce community-based linkages and governance [4].

Despite the well-documented benefits of palliative and hospice care, several barriers continue to hinder their widespread access, limiting the ability of individuals to fully benefit from these services. One prominent barrier identified in the literature is inadequate knowledge and awareness among the general public [5-7]. For example, a Canadian study highlighted persistent stigmas and misconceptions surrounding palliative care among the general public, emphasizing the necessity of targeted public education initiatives to address these knowledge gaps [8]. Similarly, research conducted in South Korea has emphasized the importance of addressing cultural barriers and enhancing public awareness to improve acceptance and use of palliative and hospice care [9]. Therefore, clearly identifying the information needs, prevalent misconceptions, and primary concerns of the general public is essential for developing effective educational materials and public awareness campaigns [10].

Modern health care information consumption has shifted significantly toward web-based platforms. The popularity of health-related podcasts, YouTube channels, blogs, and web-based communities dedicated to wellness is clear evidence of growing public interest. Moreover, many individuals actively use these web-based resources to gather health-related information, supplementing the advice they receive from health care professionals, and even seek information from other web-based platform users. For instance, in the United States, one in 3 people has attempted self-diagnosis through web searches [11]. Similarly, a survey conducted in Korea found that 88.7% (416/469) of respondents searched for medical information through web-based sources before visiting health care providers [12]. Furthermore, even sensitive topics such as addiction, abortion, and HIV are also openly discussed on platforms such as Reddit, where users actively engage in question-and-answer sessions or debates [13-15].

Recent literature underscores the importance of understanding public perceptions, including misconceptions and stigma, to effectively promote the acceptance and use of palliative care services [5-7]. In response, several studies have examined how these perceptions are expressed in social media and survey data. For example, one study analyzing social media posts during and after the COVID-19 pandemic found that palliative care was increasingly associated with fear, uncertainty, and stress, and that misconceptions about its goals and practices rose significantly during this period [16]. In contrast, an open-ended survey of 199 German-speaking adults in Switzerland revealed generally positive views, with palliative care commonly linked to interprofessional collaboration and supportive care [17].

While qualitative methods such as interviews or focus groups yield in-depth insights, they are often resource-intensive and limited in scope. Analyzing text from web-based platforms offers a scalable alternative, enabling researchers to capture broader patterns in public experiences, informational needs, and sentiment. Despite this potential, a recent systematic review of palliative care research using natural language processing techniques revealed that only 3.6% (3/82) of studies analyzed social media data [18]. This suggests that the use of web-based user-generated content, such as posts from social media, remains underused. Moreover, previous analyses, such as a Korean study on global research trends in palliative care based on academic literature [19], have not sufficiently addressed real-world public perceptions. Applying natural language processing, topic modeling, and sentiment analysis on user-generated web-based texts can help identify key concerns, main themes, and emotional responses related to palliative and hospice care from the public’s point of view.

Naver, a major search engine in South Korea, offers various services that are easily accessible to the public. The data generated on this platform have been used in various forms of research [20,21]. Among its many services, Naver Knowledge iN is a popular web-based public forum where users can share their concerns and perceptions and receive responses from others. Health-related queries are among the most common on this platform, making it an excellent resource for understanding public concerns regarding palliative and hospice care.

Therefore, this study analyzed posts from the general public on Naver Knowledge iN to provide deeper insights into the public perceptions, information needs, and sentiments regarding palliative and hospice care. Using natural language processing techniques, this study aimed to (1) identify the key topics, needs, and inquiries raised by the public; (2) examine the emotional expressions embedded in those texts; and (3) explore temporal trends in public discourse on palliative and hospice care over the past 2 decades. The findings of this study can inform targeted public awareness campaigns, the development of educational resources, and policy initiatives that better address the public’s needs and perceptions of palliative and hospice care.

## Methods

### Data Collection and Screening

The dataset for this study was collected from Naver Knowledge iN, where users post questions and receive answers from the public. Each entry on this platform consists of a user-generated question and one or more community-generated answers. As the primary aim of this study was to examine public perceptions, concerns, and information-seeking behaviors regarding palliative and hospice care, we included only the original question posts, which reflect users’ initial intentions and perspectives. Answers were excluded because they predominantly contained brief institutional referrals or promotional content (eg, advertising health care services or insurance), which were not aligned with the analytical focus of the study.

To retrieve relevant data, we used keywords such as “hospice,” “palliative care,” “palliative medicine,” “advance care planning,” and “end-of-life care.” Posts from the platform’s launch in 2002 up to June 30, 2024, were collected using Python’s BeautifulSoup library for web crawling. The extracted data were stored in a structured format that included the title, body text, and date of creation. In cases where the body text was missing, the title was used as a substitute, as it typically conveyed the user’s central inquiry.

Social media texts, such as those found on Naver Knowledge iN differ from formal medical documentation or academic articles. They often include informal language, emotive expression, and inconsistent grammar [22,23]. These characteristics required customized preprocessing procedures, such as duplicate removal, noise filtering, and lexical normalization [22,24]. This process begins with a thorough understanding of the data source, which is crucial for effective data cleaning in text-mining research [25].

In topic modeling studies, ensuring that datasets contain relevant content is critical for accurately identifying latent themes. During preprocessing, duplicate and null entries were removed from the collected dataset. Notably, some Naver Knowledge iN search results included posts where relevant keywords appeared only in the answer, rather than in the main body of the question. For example, a text such as “What certification should I pursue?” could be included if the answer mentioned “hospice nurse,” even if the main body of the question had no relevance to palliative care. To address this, we applied explicit inclusion and exclusion criteria to ensure the thematic relevance and analytical integrity of the corpus. Inclusion criteria required the presence of keywords directly related to (1) palliative or hospice care concepts, (2) specific diseases or conditions often requiring such care, or (3) life-sustaining treatment procedures. To capture diverse linguistic expressions, we used n-gram extension and ChatGPT-based term augmentation, subsequently reviewed and validated by subject-matter experts.

In contrast, exclusion criteria were derived through keyword frequency analysis and manual screening. Posts were excluded if they contained relevant keywords in non-health-related contexts, such as student assignments, pet care, dermatological concerns, academic or career guidance, insurance advertisements, or fictional writing. A complete list of these criteria is available in the Multimedia Appendix 1. Notably, due to the morphological complexity and contextual nuances of the Korean language, the n-gram and terms generated by artificial intelligence (AI) are retained in their original Korean form to preserve semantic integrity.

### Temporal Trend Analysis

To analyze temporal patterns, we extracted the year from each post’s creation date and calculated annual frequencies for each identified topic and sentiment. Because the data for 2024 were incomplete, we estimated full-year values using proportional scaling based on available months. Temporal trends were visualized with line plots, enabling examination of how public discussions evolved in response to major societal and policy events related to palliative and hospice care.

### Contextualized Topic Modeling

#### Overview

We used contextualized topic modeling (CTM) to uncover the latent topics within the corpus. CTM combines traditional word-count methods with advanced language models that understand word meaning based on context. This allows the analysis to not only identify frequently discussed words, but also to group together related ideas even when different words are used, helping uncover more accurate and meaningful topics within the text [26].

#### Preprocessing

To prepare the text data for analysis, tokenization and Part-of-Speech tagging were performed using the MeCab-ko tokenizer [27], a tool specifically designed for Korean language processing. This tokenizer splits sentences into individual word units (tokens) and identifies each word’s grammatical role (eg, noun, verb, and adjective). We first extracted only nouns from the text to focus on meaningful keywords and removed any words that appeared fewer than 5 times to eliminate noise. Additionally, we filtered out commonly used words (stopwords) in both Korean and English, which typically do not carry substantial meaning (eg, “the,” “is,” and “this”). We also applied a method to detect and preserve frequently used word pairs (bigrams), such as “hospice center,” as single units to better capture important concepts expressed across multiple words.

#### TF-IDF Embedding

Term frequency-inverse document frequency is a method that assigns higher weights to words that appear frequently in individual documents but are less common across the entire documents. This technique was used to generate a numerical representation for each document, helping to capture the important terms across the corpus [28]. To reduce dimensionality while retaining essential information, we limited vocabulary size to the 3000 most frequently occurring terms.

#### Contextual Embedding

To capture sentence-level meaning, we used a pretrained sentence bidirectional encoder representations from transformers (BERT) model (xlm-r-100langs-bert-base-nli-stsb-mean-tokens) that supports 100 languages, including Korean [29]. This model was fine-tuned using the standard natural language inference and semantic textual similarity-B datasets to generate contextual embeddings for each document. By converting each sentence into a numerical representation based on its overall meaning, the model allows for a nuanced understanding of semantically similar expressions, even when different words are used.

#### Selecting Optimal Topic Model

To identify latent topics in the text, we combined both the term frequency-inverse document frequency vectors and contextual embeddings. We trained multiple models with varying numbers of topics (ranging from 4 to 15) and training durations (epochs, ranging from 10 to 50) to find the best-performing model. Model quality was assessed using coherence scores, which evaluate how semantically related the top words in each topic are. To ensure topic diversity and reduce redundancy, we also examined inverted Rank-Biased Overlap values [30]. Finally, the selected model was manually reviewed to confirm the interpretability and thematic relevance of the identified topics.

### Multiclass Sentiment Analysis

To examine the emotional distribution in web-based posts related to palliative and hospice care and to explore the relationship between topics and emotions, we conducted a multiclass sentiment analysis using the Korean bidirectional encoder representations from transformers (KoBERT) model [31]. By leveraging the KoBERT model’s ability to capture the contextual nuances of texts, we acquire a more refined analysis than traditional binary sentiment classification, which typically divides sentiments into positive and negative categories. Polarity determination, widely used for opinion analysis, has often been applied in areas such as health policy or institutional evaluation [32,33]; however, our multiclass sentiment analysis allows us to uncover individuals’ varied emotional expressions in palliative and hospice care discussions.

The dataset used for developing the model was constructed by merging 3 publicly available Korean sentiment corpora from AIHub. The final dataset included 146,567 texts labeled across 7 emotion categories: “neutral” (n=48,616), “sadness” (n=27,517), “anger” (n=19,710), “anxiety” (n=15,999), “happiness” (n=14,406), “disgust” (n=5649), and “embarrassment” (n=14,670). During model development, the “anger” and “disgust” categories were merged into a single category called “hostility” to reduce confusion due to overlapping expressions. The final model, therefore, predicted 6 emotion classes. We randomly partitioned the dataset into training (75%) and testing (25%) sets. For model architecture, we adopted a BERTClassifier, which consists of the BERT model followed by a linear classification layer. Each sentence was first converted into tokens using a BERT tokenizer, which splits text into smaller linguistic units suitable for input into the model. To ensure uniform input length, all sequences were truncated or padded to a maximum of 64 tokens.

To address class imbalance, we applied Focal Loss, which adjusts the contribution of each class by introducing 2 hyperparameters: alpha, which is calculated dynamically based on class frequencies to assign higher weights to underrepresented classes, and gamma, which focuses learning on incorrectly predicted samples [34]. This approach significantly improves the model’s performance for minority classes. Training was conducted for 12 epochs with a batch size of 16. Throughout the training process, key performance metrics (accuracy, precision, recall, and *F*_1_-score) were tracked for both the training and testing sets. The final model was selected based on the highest test accuracy.

**Figure 1:**
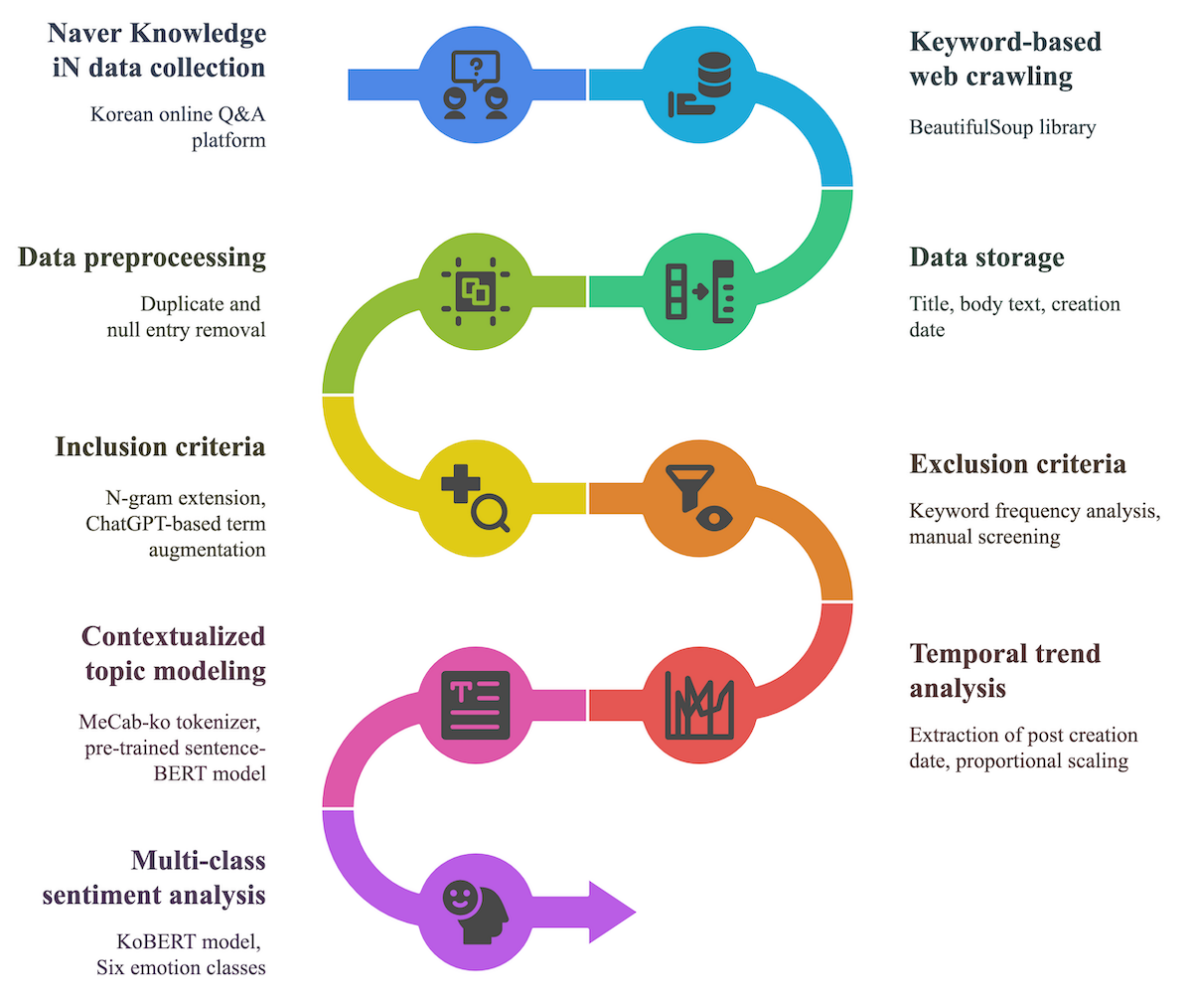
Overview of the data collection, processing, and analysis workflow. BERT: bidirectional encoder representations from transformers.

All preprocessing and analyses were conducted using Python. A summary of the overall data processing and analysis workflow is provided in Figure 1.

### Ethical Considerations

This study received exemption from review by the institutional review board at Seoul National University (institutional review board number E2407/001-003). All data used in this study were publicly available posts collected from Naver Knowledge iN. In accordance with Naver’s policy, users are explicitly advised not to include personally identifiable information in their posts. Additionally, during preprocessing, we applied term frequency thresholds to remove rare terms and conducted manual screening to exclude any entries that could potentially reveal personal identity. As a result, all analyzed data were deidentified and did not contain information that could be used to identify individual users.

## Results

### Data Characteristics

**Figure 2:**
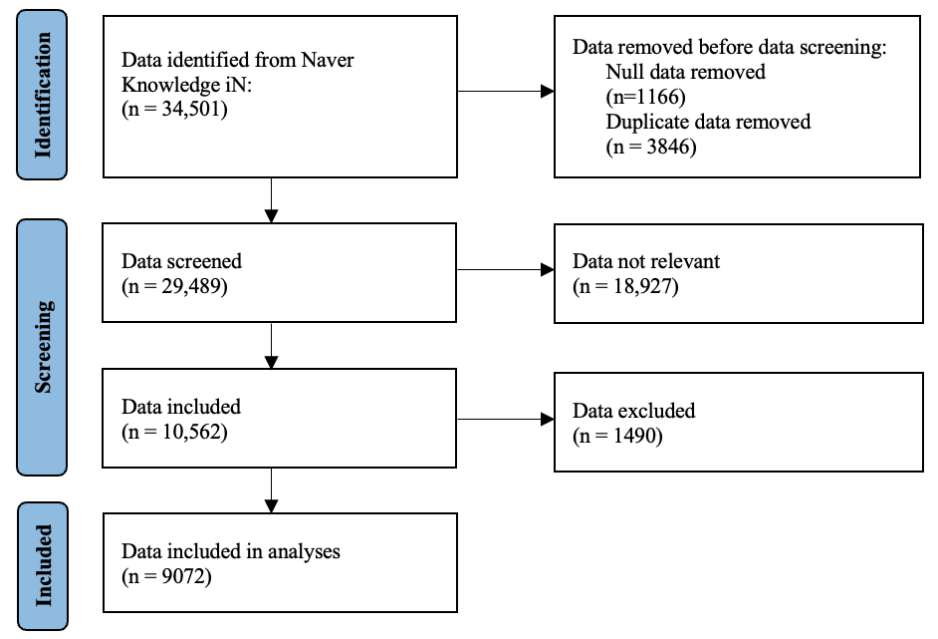
Flow diagram of data identification, screening, and inclusion process for palliative care and hospice-related posts collected from Naver Knowledge iN.

A total of 34,501 posts related to palliative and hospice care were collected from Naver Knowledge iN. After removing the null and duplicate entries, 29,489 posts were screened for relevance. Ultimately, 9072 posts were included in the final analysis after excluding irrelevant content (Figure 2). The token length distribution of the dataset had a mean token length of 177.01, with a standard deviation of 280.45. The IQR was 143.25, and the 50th percentile token length was 107.00.

### Temporal Trends in Web-Based Perspectives on Palliative and Hospice Care

**Figure 3:**
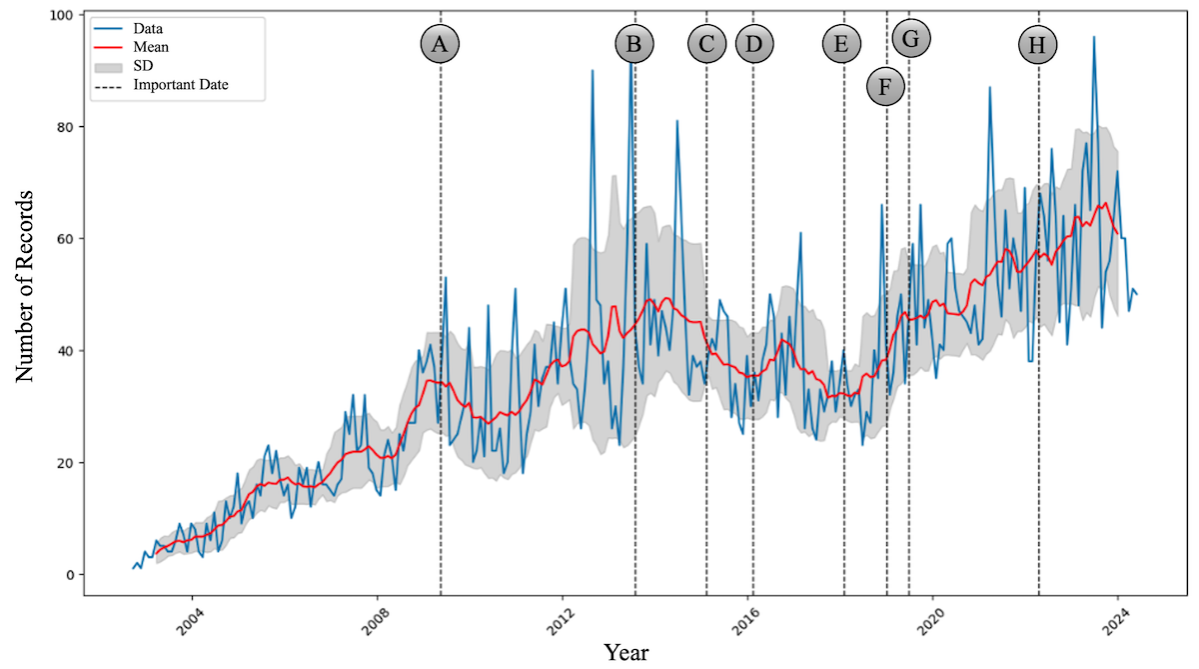
Temporal dynamics of dataset growth and important events. (A) May 2009, Kim Grandmother’s Case: the Supreme Court ruled that if a patient is medically deemed to have no chance of recovery, artificial life support can be withdrawn to respect the patient’s wishes. (B) July 31, 2013, the National Bioethics Committee recommended institutionalizing a system for life-sustaining treatment decisions. (C) February 2015, designation of a supporting agency for the National Bioethics Committee. (D) February 2016, the “Act on Decisions on Life-Sustaining Treatment for Patients in Hospice and Palliative Care or at the End of Life” (hereafter referred to as the “Life-Sustaining Treatment Decisions Act”) was enacted. (E) February 2018, implementation of the life-sustaining treatment decisions system. (F) January 7, 2019, beginning of the issuance of advance directive registration certificates for life-sustaining treatment decisions. (G) June 25, 2019, the Ministry of Health and Welfare established the “1st Comprehensive Plan for Hospice and Life-Sustaining Treatment (2019-2023).” (H) April 21, 2022, announcement of the application of regular reimbursement rates for decisions to discontinue life-sustaining treatment.

Figure 3 illustrates the temporal growth of the dataset from 2002 to 2024, with significant events related to life-sustaining treatment decisions marked along the timeline. Over time, a steady increase in the number of records was observed, with several key legal and institutional milestones potentially influencing this trend.

The first notable event, Kim Grandmother’s Case in May 2009 (Figure 3A), coincided with a gradual increase in the growth of the dataset. This event, in which the Supreme Court ruled in favor of respecting patients’ wishes to withdraw life-sustaining treatment, may have sparked an increased public discourse on end-of-life care.

In July 2013 (Figure 3B), the National Bioethics Committee’s recommendation to institutionalize life-sustaining treatment decisions corresponded to a period of steady data accumulation. This growth continued, peaking after the issuance of advance directive registration certificates in January 2019 (Figure 3F) and the establishment of the “1st Comprehensive Plan for Hospice and Life-Sustaining Treatment” in June 2019 by the Ministry of Health and Welfare (Figure 3G). Finally, in April 2022 (Figure 3H), the announcement of regular reimbursement rates for decisions to discontinue life-sustaining treatment correlates with a sharp increase in data volume.

### Contextualized Topic Model

**Figure 4:**
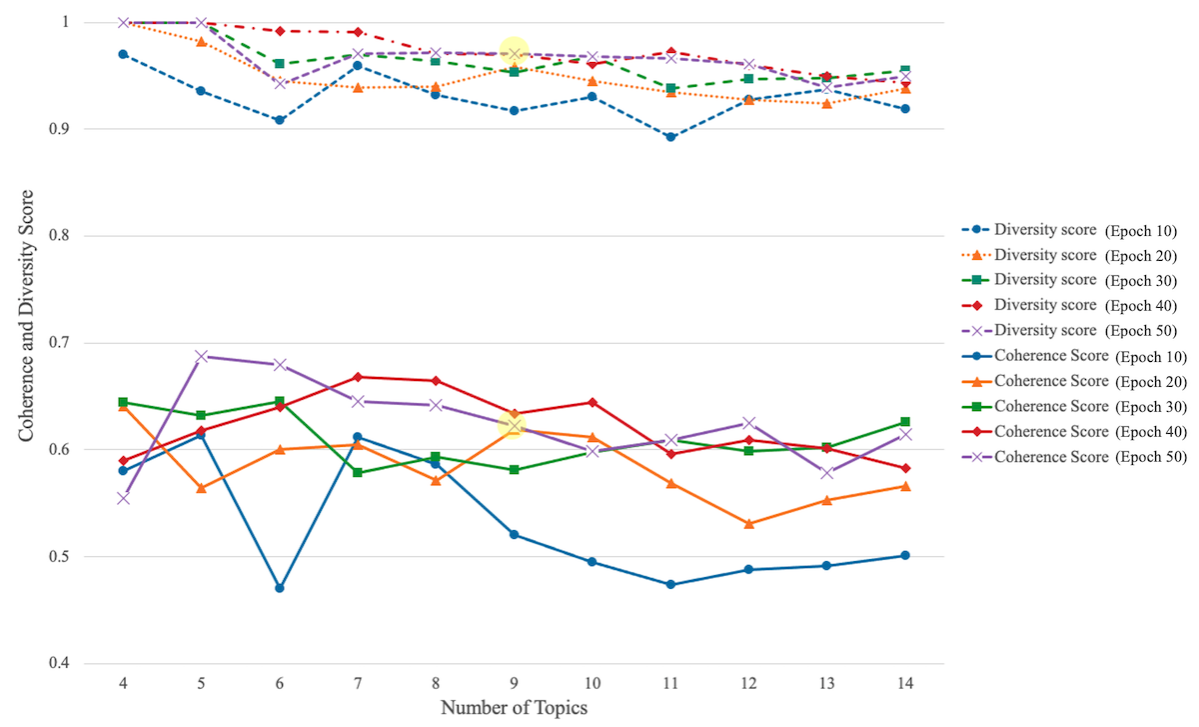
Coherence and diversity scores across several topics and epochs for contextualized topic modeling. Yellow highlights indicate the final selected model.

To identify the key topics of interest from 9072 web-based posts related to palliative and hospice care, we performed a CTM while varying the number of epochs and topics. The diversity scores for the topics consistently remained above 0.9, indicating high topic diversity across the models. For the coherence scores, the researchers closely examined models with a coherence score greater than 0.6. Based on this expert review, a model with 9 topics trained over 50 epochs was selected as the final CTM because it balanced diversity, coherence, and interpretability (Figure 4).

Table 1 presents the results of the CTM for 9072 web-based perspectives related to palliative and hospice care that identified 9 distinct themes. The largest theme, “emotional and psychological support,” accounted for 15.95% (1447/9072) of the data, followed by “ethical and legal concerns” (1170/9072, 12.90%) and “medical care in hospitals” (1105/9072, 12.18%). Other identified themes included “nutrition management” (1096/9072, 12.08%), “disease treatment outcomes and prognosis” (1078/9072, 11.88%), “financial issues” (908/9072, 10.01%), “symptom management” (904/9072, 9.96%), “administrative and volunteer services” (832/9072, 9.17%), and “health insurance” (532/9072, 5.86%). These results highlight a broad range of concerns, from emotional support to legal and financial issues, within the context of palliative and hospice care.

**Table 1:** Themes identified in social media perspectives on palliative and hospice care.

Themes	Keywords			Proportions
	English (translation)	Korean (original)		
Emotional and Psychological Support	Love, Tears, Memories, Sound, Last, God, Life, Story, Appearance, Death, Emotion, Happiness, Prayer, Regret, Pain, Marriage, Face, Pronouncement, Religion, Church	사랑, 눈물, 기억, 소리, 마지막, 하나님, 인생, 이야기, 모습, 죽음, 감정, 행복, 기도, 후회, 고통, 결혼, 얼굴, 선고, 종교, 교회	0.159502	
Ethical and Legal Concerns	Life-sustaining treatment, Brain death, Euthanasia, DNR^a^, Refusal of life-sustaining treatment, Withdrawal of life-sustaining treatment, Ethics, Life-sustaining medical care, Death with dignity, Brain death, Rebuttal, Die, Interpretation, Agreement, Opposition, Active, Rationale, Device, Consent form, Prohibition	연명 치료, 뇌사, 안락사, dnr, 연명 치료 거부, 연명 치료 중단, 윤리, 연명 의료, 존엄사, 식물 인간, 반박, die, 해석, 찬성, 반대, 적극, 근거, 장치, 동의서, 금지	0.128968	
Medical Care in Hospitals	Hospital, Long-term care hospital, Treatment, Doctor, Situation, Pain, Seoul^b^, Hospitalization, Meal, Examination, Anticancer, Surgery, Anticancer treatment, Pain, Discharge, Possibility, Abnormality, Painkiller, Nursing home, Patient	병원, 요양 병원, 치료, 의사, 상황, 통증, 서울, 입원, 식사, 검사, 항암, 수술, 항암 치료, 고통, 퇴원, 가능, 이상, 진통제, 요양원, 환자	0.121803	
Nutrition Management	Mushroom, Efficacy, Food, Dietary products, Effect, Consumption, Vegetables, Stomach cancer, AIDS, Fruits, Diabetes mellitus, Purchase, Leukemia, Alzheimer disease, Prevention, Tobacco, Habit, Product, Oral cancer, Diabetes	버섯, 효능, 음식, 식품, 효과, 복용, 야채, 위암, 에이즈, 과일, 당뇨병, 구입, 백혈병, 알츠하이머, 예방, 담배, 습관, 제품, 구강암, 당뇨	0.120811	
Disease Treatment Outcomes and Prognosis	Surgery, Metastasis, Anticancer treatment, Cure, Survival, Breast cancer, Early stage, Recurrence, Cancer cells, Examination, Probability, Treatment, Lung cancer, Discovery, Lymph nodes, Anticancer, Radiation therapy, Result, Possibility, CT^c^ scan	수술, 전이, 항암 치료, 완치, 생존, 유방암, 초기, 재발, 암세포, 검사, 확률, 치료, 폐암, 발견, 임파선, 항암, 방사선 치료, 결과, 가능, ct	0.118827	
Financial Issues	Property, Divorce, Ownership, Contact, Application, Inheritance, Loan, Apartment, Report, Donation, Hospital bills, Renunciation of inheritance, Phone call, Caregiving, Real estate, Debt, Lawsuit, Rent, Document, Tax	재산, 이혼, 명의, 연락, 신청, 상속, 대출, 아파트, 신고, 증여, 병원비, 상속 포기, 전화, 간병, 부동산, 채무, 소송, 월세, 서류, 세금	0.100088	
Symptom Management	Hospital, Treatment, Anticancer treatment, Surgery, Doctor, Examination, Pain, Symptoms, Teacher (doctor), Metastasis, Anticancer, Meal, Terminal liver cancer, Painkiller, Seoul, Long-term care hospital, Pain, Discharge, Abnormality, Hospitalization	병원, 치료, 항암 치료, 수술, 의사, 검사, 통증, 증상, 선생, 전이, 항암, 식사, 간암 말기, 진통제, 서울, 요양 병원, 고통, 퇴원, 이상, 입원	0.099647	
Administrative and Volunteer Services	Hospice, Hospice nurse, Hospice ward, Nurse, Specialist nurse, Volunteering, Advance directive for life-sustaining treatment, Hospice hospital, Social welfare, Activity, Nursing, Resource, Registration, Role, Medical care, Yongin^b^, Writing, Graduate school, Institution, Internship	호스피스, 호스피스 간호사, 호스피스 병동, 간호사, 전문 간호사, 봉사, 사전 연명 의료 의향서, 호스피스 병원, 사회 복지, 활동, 간호, 자원, 등록, 역할, 의료, 용인, 작성, 대학원, 기관, 실습	0.091711	
Health Insurance	Insurance, Disease, Coverage, Recommendation, Death, Diagnosis, Renewal, Premium, Insurance payment, Illness, Health insurance, Dementia, Product, Whole life insurance, Life, Contract, Hospitalization per diem, Medical expenses, Fire (insurance), Planning	보험, 질병, 보장, 추천, 사망, 진단, 갱신, 보험료, 보험금, 질환, 건강 보험, 치매, 상품, 종신 보험, 생명, 계약, 입원 일당, 의료비, 화재, 설계	0.058642	

^a^DNR: do-not-resuscitate.

^b^Name of the city in South Korea.

^c^CT: computed tomography.

**Figure 5:**
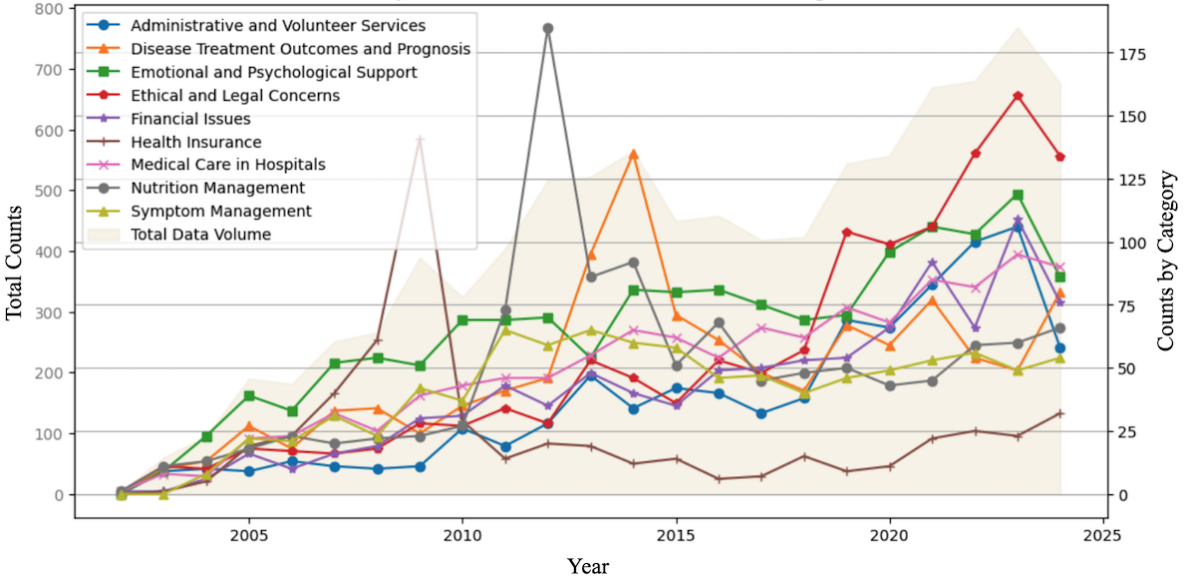
Annual topic distribution and data volume in social media perspectives on palliative and hospice care (2002-2024). Also, 2024 values are extrapolated.

Figure 5 illustrates the annual distribution of the topics identified through the CTM analysis, displayed as line graphs, along with the total volume of data per year, shown as a shaded area plot. Notably, topics such as “ethical and legal concerns” and “medical care in hospitals” have demonstrated considerable peaks in recent years, suggesting growing public interest in these areas. In contrast, topics such as “nutrition management,” “symptom management,” and “health insurance” fluctuated around the 2010s but have maintained a relatively lower presence in the 2020s.

Throughout the study period, “emotional and psychological support” consistently remained among the most frequently discussed topics. Perspectives on “disease treatment outcomes and prognosis,” “financial issues,” and “administrative and volunteer services,” topics related to patients’ circumstances, financial matters, and administrative processes rather than direct care, were moderately represented.

### Multiclass Sentiment Analysis

**Figure 6:**
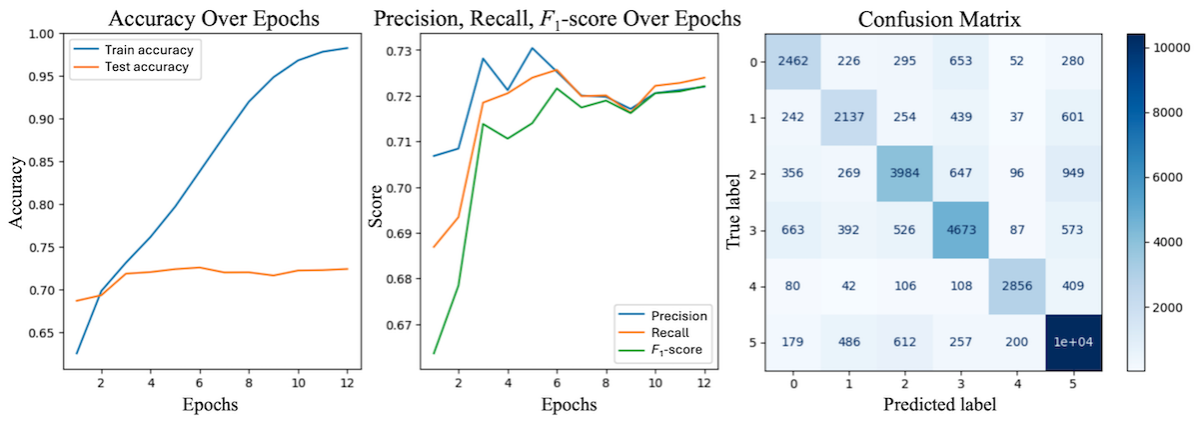
Performance metrics of the multiclass sentiment analysis model: Accuracy, Precision, Recall, F1-score (harmonic mean of precision and recall), and Confusion Matrix (label: 0 = anxiety, 1 = embarrassment, 2 = hostility, 3 = sadness, 4 = happiness, and 5 = neutral).

Figure 6 shows the performance of the multiclass sentiment analysis model across several key metrics. The left plot shows accuracy trends over 12 epochs, where the train accuracy (blue line) steadily improved to nearly 98%, whereas the test accuracy (orange line) stabilized at approximately 72%. The middle plot provides an overview of the precision, recall, and *F*_1_-score. Precision (blue) peaks early but slightly decreases in later epochs, whereas recall (orange) gradually improves and converges with precision around epoch 8. The *F*_1_-score (green) represents the balance between Precision and Recall, followed by similar trends, and stabilized at approximately 72% toward the end of training. The best model performance was observed at epoch 5, with an accuracy of 0.72, precision of 0.73, recall of 0.72, and an *F*_1_-score of 0.71. The plot on the right presents a Confusion Matrix depicting the true versus predicted label distribution across the 6 sentiment classes for the best-performing model. It indicates strong performance in certain classes, such as “neutral” (label 5), which shows the highest number of correct predictions.

**Figure 7:**
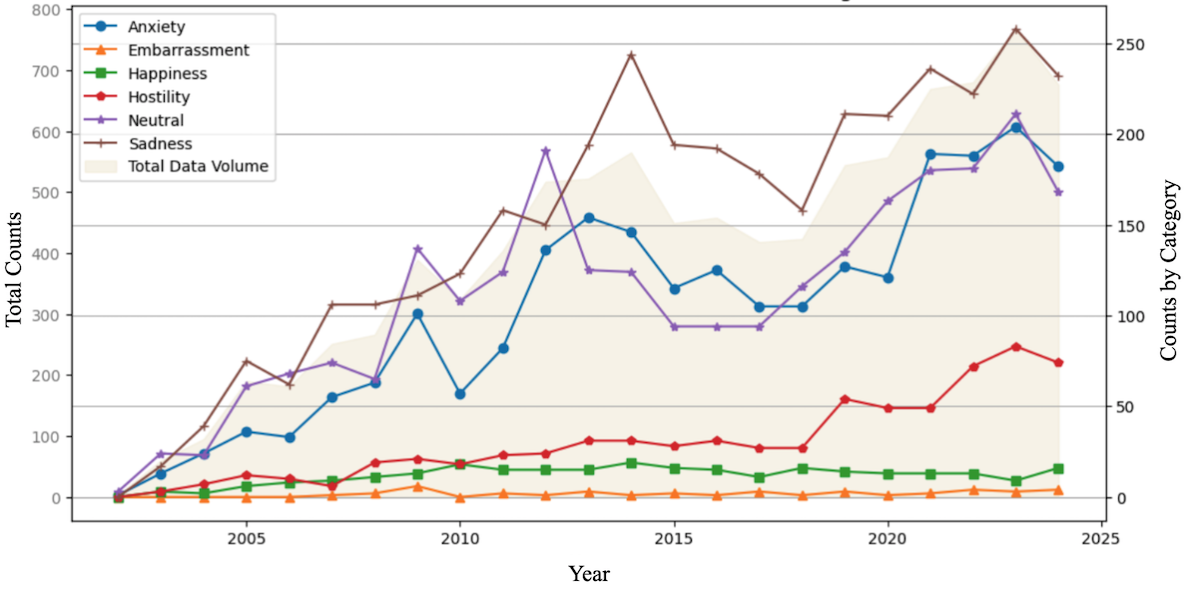
Annual sentiment distribution and data volume in social media perspectives on palliative and hospice care (2002-2024). Also, 2024 values are extrapolated.

Figure 7 illustrates the annual distribution of sentiments identified in perspectives related to palliative and hospice care using the best-performing multiclass sentiment analysis model identified in the previous stage. “neutral” and “sadness” sentiments dominate the dataset, with “sadness” showing a marked increase from 2015 onwards, peaking in recent years. “anxiety” also exhibits a steady rise over time. In contrast, sentiments such as “embarrassment,” “happiness,” and “hostility” maintain a relatively lower and stable distribution across the time frame.

### Relationships Between the Topics and the Predicted Sentiments

**Figure 8:**
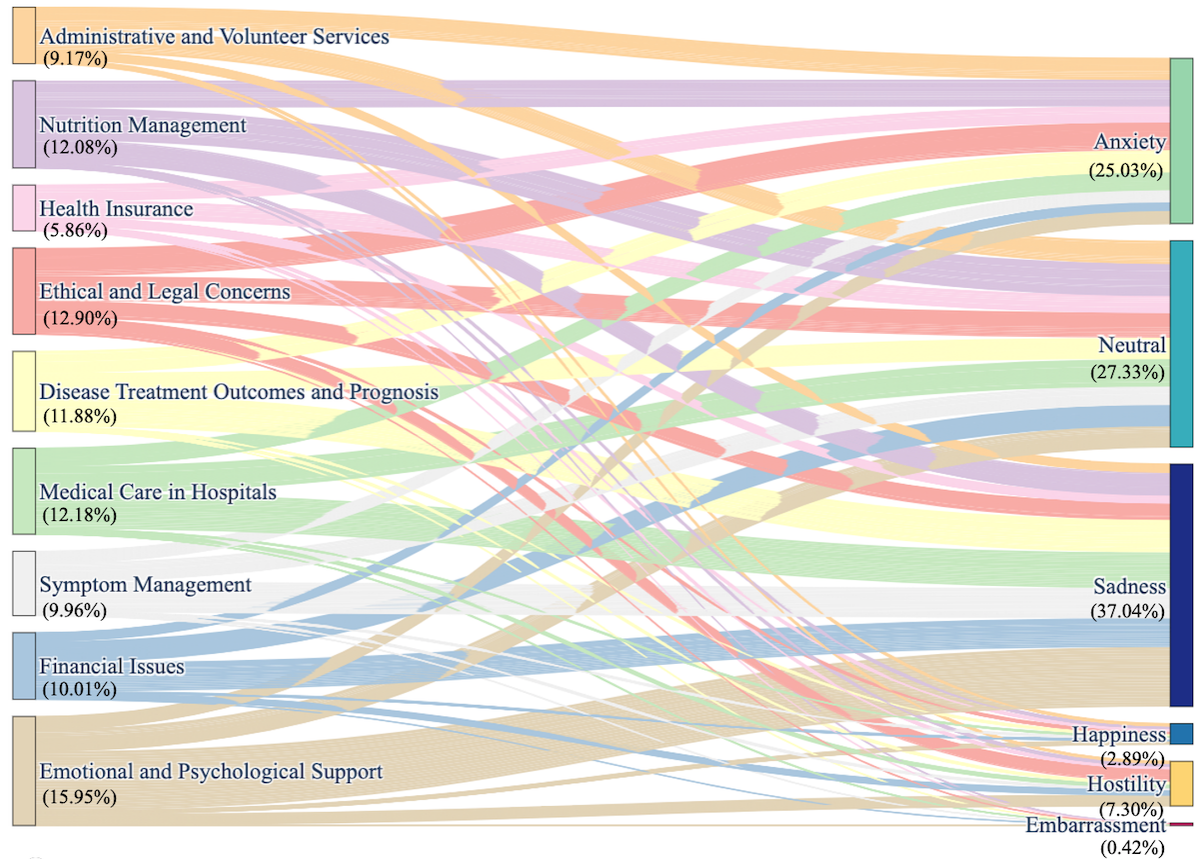
Topic-to-sentiment flow in social media perspectives on palliative and hospice care.

Figure 8 shows the relationship between the topics identified through the CTM and the sentiments predicted by the multiclass sentiment analysis model. A notable observation was that the majority of topics, such as “emotional and psychological support,” “disease treatment outcomes and prognosis,” “medical care in hospitals,” and “symptom management,” were predominantly associated with “sadness.” All 9 topics exhibit similar connections to the “neutral” sentiment, while topics such as “administrative and volunteer services” and “nutrition management” show the strongest association with “anxiety.”

## Discussion

### Principal Findings

This study demonstrates that public interest in palliative and hospice care, as captured through web-based discourse, tends to rise sharply in response to major policy changes and high-profile legal cases. These spikes suggest that regulatory and institutional developments, such as the establishment of national advance directive systems or legislation governing life-sustaining treatment, function as societal turning points that prompt individuals to seek information and engage with end-of-life care issues. This pattern is consistent with prior findings by Huemer et al [35], who showed that legislative changes across Austria, Germany, and Switzerland significantly influenced public search behaviors related to palliative care, often redirecting attention away from controversial practices such as euthanasia toward more supportive care models.

The role of media in catalyzing these shifts cannot be overstated. In South Korea, the “Kim Grandmother’s Case” served as a defining moment that not only raised public awareness but also led to institutional and legal reforms. Media coverage, particularly investigative reports and emotionally resonant narratives, can demystify palliative care, correct misconceptions, and stimulate public discourse [36]. Yet, despite these impactful events and ongoing promotional efforts, national awareness of palliative and hospice care remains relatively low at 35.7% (4676/13,546) [9], suggesting that current outreach may not be sufficient to shift cultural norms or drive meaningful behavioral change.

Given these dynamics, social media platforms offer a critical, real-time lens through which evolving public concerns can be monitored and addressed. The clear temporal spikes observed in our analysis illustrate that social discourse is sensitive to policy events and ethical debates. This highlights the need for responsive communication strategies that can align with these moments of heightened attention. By leveraging timely messaging, expanding public education, and integrating awareness campaigns with broader policy initiatives, stakeholders can foster more informed, stigma-free conversations about palliative care. Doing so may ultimately improve uptake of services and better support patients and families navigating end-of-life decisions.

Building on these observations, it is important to consider the specific topics that have gained prominence in public discourse. In particular, topics such as “ethical and legal concerns,” “administrative and volunteer services,” “disease treatment outcomes and prognosis,” and “medical care in hospitals” have demonstrated considerable increases in recent years, suggesting growing public interest and need for information. Numerous studies have emphasized the importance of providing palliative and hospice care information early in the course of illness [37,38]. However, a systematic review revealed that more than half of the patients remain unaware of such services [39]. To address this gap, targeted promotional strategies and public education materials should be developed that speak not only to patients and families but also to the broader public [39]. A more inclusive and tailored communication approach may help meet diverse informational needs and promote earlier, more proactive engagement with palliative and hospice care.

Topics related to “nutrition management” were prominent, indicating heightened interest in dietary considerations within palliative care, which aligns with East Asian caregiving traditions that emphasize high-quality, condition-specific food as a key component of care [40]. This cultural focus may also reflect the influence of eastern medicine, which emphasizes maintaining health through diet and natural remedies [41].

Meanwhile, the “ethical and legal concerns” theme, encompassing keywords such as “euthanasia,” “life-sustaining treatment,” “DNR (Do-Not-Resuscitate),” “refusal of life-sustaining treatment,” and “withdrawal of life-sustaining treatment,” reflects ongoing social discussions in Korea regarding the discontinuation of futile life-sustaining treatments. This topic can be viewed with particular consideration to this study’s findings on temporal trends in web-based palliative and hospice care perspectives. In particular, the 1997 “Boramae Hospital Case” (which preceded our data analysis period) and the 2008 “Kim Grandmother’s Case” drew substantial public attention to the issue of withdrawal of life-sustaining treatment in Korea, ultimately prompting the creation of related legislation. The “Boramae Hospital Case” involved an intensive care unit patient dependent on mechanical ventilation whose family requested discharge against medical advice due to the treatment costs; the patient subsequently died at home. Another family member sued both the requesting family and the medical staff, and the court found the medical team guilty of aiding homicide through inaction [42]. Following this case, conflicts arose between health care providers and families regarding continued life-sustaining treatment for patients with no chance of recovery. These issues became starkly apparent in the 2008 “Kim Grandmother’s Case,” in which the family of an older woman in a persistent vegetative state requested withdrawal of mechanical ventilation, citing the patient’s previously expressed wish for a natural death. However, the earlier “Boramae Hospital Case” influenced the medical team’s refusal. Eventually, the family filed a constitutional complaint, leading to Korea’s first legal precedent permitting the withdrawal of life-sustaining treatment [43].

Despite these legal milestones, withdrawal of treatment remains infrequent in Korea (550/54,699, about 1%) compared with countries like Sweden (7865/12,072, 65.2%) and the global average (42.3%) [44-46]. Contributing factors include persistent misconceptions about treatment cessation, limited public awareness, and low completion rates of advanced care plans among adults, and gaps in the official system for creating and registering these plans [9,47,48]. Nevertheless, the frequent appearance of these issues in web-based discourse reflects a growing desire for compassionate, dignified death. Therefore, we propose integrating palliative and hospice care education into public and school curricula, providing a structured means of preparing for the universal experience of death while emphasizing the value of end-of-life care.

Financial concerns are prominently reflected in public discourse on palliative and hospice care, with approximately 16% (1440/9072) of analyzed posts referencing financial burdens or health insurance, comparable in frequency to expressions of emotional distress. Previous research highlights high treatment costs, limited reimbursement, and income loss from prolonged caregiving as critical barriers to care access [49-51]. For families facing financial strain, home-based palliative care can offer a dignified and more affordable alternative to institutional settings [52]. Indeed, studies have shown that early integration of palliative and hospice services contributes to substantial health care cost reductions [53,54].

In South Korea, the National Health Insurance Service reduces copayments to 0%-10% for patients with terminal cancer, cardiovascular, cerebrovascular, rare, and intractable diseases, significantly lower than the standard 20% for inpatient and 30%-60% for outpatient care. Home-based palliative care, which costs only 9% of equivalent inpatient services (225,688 KRW [US $192.24] vs 2,481,479 KRW [US $2113.70] per week; conversion based on KRW 1174 per US $1 in September 2020), offers significant savings [55]. The long-term care insurance system provides in-home care services for eligible older adults, and the Comprehensive Nursing Service System supports hospital-based care without requiring private caregivers. In addition, the National Responsibility Policy for Dementia offers financial subsidies for dementia caregivers. However, existing policies fall short in several respects. The 90-day unpaid family care leave permitted under the Equal Employment Opportunity and Work-Family Balance Assistance Act lacks wage replacement, thereby exacerbating financial burdens. Moreover, many indirect nonmedical expenses, such as transportation or respite care, remain uncovered.

Educational materials should not only emphasize the cost-saving benefits of palliative and hospice care but also provide detailed information on available financial support. Policy efforts must expand to include paid family care leave and promote equitable access to home-based services. In particular, coordination with existing public health initiatives, such as home-visiting nursing services via community health centers or long-term care insurance programs, could enhance outreach, education, and timely identification of eligible patients in need of palliative care.

A notable finding of the sentiment analysis was the high prevalence of neutral sentiments, which were relatively evenly distributed across all identified topics. This pattern reflects the informational nature of the social media platform, Naver Knowledge iN, where users often seek factual guidance rather than emotional support. Representative questions include: “Where can I draft an advance directive for life-sustaining treatment?” and “Which mushrooms are beneficial to terminal cancer patients?”. These examples point to a strong public demand for practical, reliable, and easily accessible information related to end-of-life care. In response to this need, the development of AI-driven tools, such as large language model–based domain-specific chatbots, could offer a scalable solution. By delivering timely, accurate, and empathetic answers, such tools can help reduce informational barriers, address misconceptions, and better support both individuals directly affected by serious illness and members of the general public seeking guidance.

In contrast, “sadness” emerged as the most frequent emotion, followed by “neutral” and “anxiety.” The prominence of “sadness” reflects the emotional burden commonly associated with serious illness and end-of-life decision-making. Although the identities of individual posters remain unknown due to the platform’s anonymity, it is plausible that many posts were authored by patients, family caregivers, or individuals with personal experience of palliative care. Notably, “anxiety,” “hostility,” and “embarrassment” were closely linked to topics such as “emotional and psychological support,” “nutrition management,” and “symptom management,” suggesting frequent emotional struggles in navigating disease progression. These findings align with prior research reporting high levels of psychological distress among people facing terminal illness [56,57]. Accordingly, beyond managing physical symptoms, health care providers should prioritize emotional and psychosocial support to ensure holistic care [39,58]. Moreover, these insights indicate that educational interventions must be designed not only to deliver accurate medical information but also to meaningfully engage with the emotional and ethical concerns that shape public attitudes toward palliative and hospice care.

Despite the growing public interest in palliative care, communication between health care providers and individuals facing serious illness remains a persistent challenge [18]. Misconceptions, such as viewing palliative care as synonymous with “giving up” on curative treatment, alongside cultural taboos surrounding death, often hinder open and timely dialogue [5]. Addressing these barriers requires culturally sensitive public education and sustained efforts to reframe palliative care as a proactive and empowering choice. In clinical contexts, tools such as the supportive and palliative care indicators tool can facilitate communication by providing clinicians with structured conversation guides that support early and compassionate discussions about palliative needs [59]. Embedding such tools into clinical workflows and linking them with public education initiatives may improve patient-provider communication, reduce stigma, and promote earlier access to supportive services.

Beyond clinical tools such as the supportive and palliative care indicators tool that support provider-initiated conversations, broader cultural change is also necessary to normalize discussions about death and dying across society. In South Korea, while the concept of dignified dying (also known as well-dying) has gained limited attention in the context of an aging society, public discourse around death remains marginal. Cultural taboos rooted in Confucian and Buddhist traditions often associate such discussions with misfortune, inhibiting timely communication among health care providers, patients, and families [60,61]. A national survey of Korean adults revealed that 52.7%-84.0% rarely or never discuss death, and only 17.9%-32.2% feel confident about their end-of-life preferences [62]. To address this, public education, community engagement, and school-based death education programs should be expanded to reduce stigma and promote timely, values-based decision-making about palliative and hospice care.

### Limitations

This study has several limitations, particularly in the context of broader frameworks of serious illness and care. First, the analysis of Naver Knowledge iN posts lacked contextual details regarding the author’s background as well as clinical information such as diagnosis, disease stage, or palliative care status. These limitations stem from the anonymous and unstructured nature of the platform. As a result, the dataset may not fully reflect the diversity of serious illness experiences, including non-cancer conditions such as chronic obstructive pulmonary disease or congestive heart failure [63]. Future studies should incorporate alternative data sources that allow for the identification of author roles and longitudinal care trajectories to better account for structural and contextual influences on palliative care needs. Second, we excluded answer posts from the discussion threads during preprocessing. While many of these entries were promotional or duplicative, their exclusion may have narrowed the range of perspectives included in the analysis. Future research could explore these responses using preprocessing and filtering techniques to extract informative content. Third, although our multiclass sentiment analysis achieved robust performance (accuracy, precision, recall, and *F*_1_-score all above 0.70), class imbalance remained a challenge. For instance, low-frequency emotions such as “embarrassment” may still be underdetected [64]. This issue could be addressed in future work through advanced data augmentation techniques or by collecting larger and more diverse datasets. Fourth, the perspectives of bereaved families may have been underrepresented due to the keyword-based data collection strategy. Given that author identity could not be determined, it is likely that posts written by bereaved caregivers were unintentionally excluded. Refining search strategies and terminology to better capture bereaved experiences would improve the inclusivity and depth of future research. Last, although our approach was grounded in large-scale computational analysis, it did not examine whether individual posts referenced specific policy changes or widely mediatized cases. Future research could build on our findings by conducting a secondary analysis of a subset of our dataset, focusing on posts written by specific groups such as patients and family caregivers. Qualitative methods, such as content analysis or reflexive thematic analysis, could help uncover how these groups interpret ethically complex issues such as the withdrawal of life-sustaining treatment. Such analysis would also enrich our understanding of the narratives and concerns that are unique to these groups and how they respond to social and institutional changes.

### Conclusions

This study offers novel insights into public discourse on palliative and hospice care in South Korea by analyzing large-scale social media data. Our findings reveal that emotionally negative sentiments, particularly sadness and anxiety, are prevalent and closely tied to key concerns such as symptom management, ethical dilemmas, and financial burdens. Notably, approximately 16% (1440/9072) of posts addressed concerns related to caregiving stress or economic hardship, reflecting the multifaceted challenges of palliative care as expressed by individuals participating in web-based discussions. The prevalence of neutral, fact-seeking queries underscores a strong public demand for accessible and reliable information. At the same time, widely documented misconceptions, such as viewing palliative care as giving up on treatment, continue to hinder effective communication, reinforcing the need for clearer communication. However, the anonymous and unstructured nature of platforms like Naver Knowledge iN limits their potential for sustained, interactive dialogue. To address this gap, user-centered tools, such as culturally tailored educational materials, AI-powered chatbots, or moderated web-based forums, could provide timely and emotionally responsive support. These tools may also bridge public needs and clinical systems by identifying emerging concerns and facilitating early intervention. In parallel, policy and education initiatives, including the integration of death education into school and community curricula, are essential to normalize conversations about dying and promote informed, compassionate decision-making. By demonstrating the utility of social media analysis for capturing real-time public concerns, this study contributes to global efforts in end-of-life care advocacy, underscoring the importance of culturally responsive communication strategies and emotional support infrastructures.

## Appendix

Multimedia Appendix 1 Criteria for data inclusion and exclusion in screening.

## Abbreviations

AI: artificial intelligence
BERT: bidirectional encoder representations from transformers
CTM: contextualized topic modeling
KoBERT: Korean bidirectional encoder representations from transformers

## Data Availability

The datasets analyzed during this study are not publicly available due to personal information protection regulations and platform policies but are available from the corresponding author on reasonable request.

## References

[1] (2021). What are palliative care and hospice care?. NIH National Institute of Aging.

[2] Cohen J, Gott M (2015). Dying in place in old age: public health challenges. Palliative Care for Older People: Public Health Perspective.

[3] Meghani SH (2004). A concept analysis of palliative care in the United States. J Adv Nurs.

[4] Kim K, Kim W, Jeong NE, Yeon KS, Young CJ (2025). The national hospice policy of the 2nd comprehensive hospice and life- sustaining treatment plan (2024-2028) in the Republic of Korea. Health Policy Manag.

[5] Cai Y, Lalani N (2022). Examining barriers and facilitators to palliative care access in rural areas: a scoping review. Am J Hosp Palliat Care.

[6] Li W, Chhabra J, Singh S (2021). Palliative care education and its effectiveness: a systematic review. Public Health.

[7] McIlfatrick S, Slater P, Beck E, Bamidele O, McCloskey S, Carr K, Muldrew D, Hanna-Trainor L, Hasson F (2021). Examining public knowledge, attitudes and perceptions towards palliative care: a mixed method sequential study. BMC Palliat Care.

[8] Zimmermann C, Wong JL, Swami N, Pope A, Cheng Y, Mathews J, Howell D, Sullivan R, Rodin G, Hannon B, Moineddin R, Le LW (2021). Public knowledge and attitudes concerning palliative care. BMJ Support Palliat Care.

[9] Kim B, Lee J, Choi YS (2023). Public awareness of advance care planning and hospice palliative care: a nationwide cross-sectional study in Korea. BMC Palliat Care.

[10] Schofield G, Dittborn M, Huxtable R, Brangan E, Selman LE (2021). Real-world ethics in palliative care: a systematic review of the ethical challenges reported by specialist palliative care practitioners in their clinical practice. Palliat Med.

[11] Kuehn BM (2013). More than one-third of US individuals use the internet to self-diagnose. JAMA.

[12] Song TM, Park EJ, Lim EJ (2002). The survey of the demand for health information on the internet. J Korean Soc Med Inform.

[13] Valdez D, Mena-Meléndez L, Crawford BL, Jozkowski KN (2024). Analyzing reddit forums specific to abortion that yield diverse dialogues pertaining to medical information seeking and personal worldviews: data mining and natural language processing comparative study. J Med Internet Res.

[14] Chi Y, Chen H (2023). Investigating substance use via reddit: systematic scoping review. J Med Internet Res.

[15] Loosier PS, Renfro K, Carry M, Williams SP, Hogben M, Aral S (2022). Reddit on PrEP: posts about pre-exposure prophylaxis for HIV from Reddit users, 2014-2019. AIDS Behav.

[16] Peters J, Heckel M, Breindl E, Ostgathe C (2024). What does "Palliative" mean? sentiment, knowledge, and public perception concerning palliative care on the internet since the COVID-19 pandemic. Palliat Med Rep.

[17] Fliedner MC, Zambrano SC, Eychmueller S (2021). Public perception of palliative care: a survey of the general population. Palliat Care Soc Pract.

[18] Sarmet M, Kabani A, Coelho L, Dos Reis SS, Zeredo JL, Mehta AK (2023). The use of natural language processing in palliative care research: a scoping review. Palliat Med.

[19] Park E, Kim Y, Park CS (2017). [A comparison of hospice care research topics between Korea and other countries using text network analysis]. J Korean Acad Nurs.

[20] Husnayain A, Shim E, Fuad A, Su EC (2021). Predicting new daily COVID-19 cases and deaths using search engine query data in South Korea from 2020 to 2021: infodemiology study. J Med Internet Res.

[21] Park TH, Kim WI, Park S, Ahn J, Cho MK, Kim S (2020). Public interest in acne on the internet: comparison of search information from google trends and naver. J Med Internet Res.

[22] Ali Mustofa B, Laksito Yuly Saptomo W (2025). Use of natural language processing in social media text analysis. J Artif Intell Eng Appl.

[23] Baldwin T (2012). Social media: friend or foe of natural language processing?. https://aclanthology.org/Y12-1005/.

[24] Foufi V, Timakum T, Gaudet-Blavignac C, Lovis C, Song M (2019). Mining of textual health information from reddit: analysis of chronic diseases with extracted entities and their relations. J Med Internet Res.

[25] Orr W, Crawford K (2024). Building better datasets: seven recommendations for responsible design from dataset creators. arXiv:2409.00252.

[26] Bianchi F, Terragni S, Hovy D (2021). Pre-training is a hot topic: contextualized document embeddings improve topic coherence.

[27] SOMJANG (2023). Mecab-ko-for-Google-Colab. GitHub repository.

[28] Manning CD, Manning CD, Raghavan P, Schütze H (2008). Introduction to Information Retrieval.

[29] Reimers N, Gurevych I (2020). Making monolingual sentence embeddings multilingual using knowledge distillation.

[30] Zankadi H, Idrissi A, Daoudi N, Hilal I (2023). Identifying learners' topical interests from social media content to enrich their course preferences in MOOCs using topic modeling and NLP techniques. Educ Inf Technol (Dordr).

[31] SKTBrain (2004). Korean BERT pre-trained cased (KoBERT). GitHub Repository.

[32] Liu M, Yuan S, Li B, Zhang Y, Liu J, Guan C, Chen Q, Ruan J, Xie L (2024). Chinese public attitudes and opinions on health policies during public health emergencies: sentiment and topic analysis. J Med Internet Res.

[33] Alexander G, Bahja M, Butt GF (2022). Automating large-scale health care service feedback analysis: sentiment analysis and topic modeling study. JMIR Med Inform.

[34] Lin TY, Goyal P, Girshick R, He K, Dollar P (2017). Focal loss for dense object detection.

[35] Huemer M, Jahn-Kuch D, Hofmann G, Andritsch E, Farkas C, Schaupp W, Masel EK, Jost PJ, Pichler M (2021). Trends and patterns in the public awareness of palliative care, Euthanasia, and end-of-life decisions in 3 central European countries using big data analysis from Google: retrospective analysis. J Med Internet Res.

[36] Carrasco JM, Gómez-Baceiredo B, Navas A, Krawczyk M, García M, Centeno C (2019). Social representation of palliative care in the Spanish printed media: a qualitative analysis. PLoS One.

[37] Austin L, Luker K, Caress A, Hallett C (2000). Palliative care: community nurses' perceptions of quality. Qual Health Care.

[38] Welch LC, Miller SC, Martin EW, Nanda A (2008). Referral and timing of referral to hospice care in nursing homes: the significant role of staff members. Gerontologist.

[39] Lorenz KA, Lynn J, Dy SM, Shugarman LR, Wilkinson A, Mularski RA, Morton SC, Hughes RG, Hilton LK, Maglione M, Rhodes SL, Rolon C, Sun VC, Shekelle PG (2008). Evidence for improving palliative care at the end of life: a systematic review. Ann Intern Med.

[40] Martinson IM, Leavitt M, Liu C, Armstrong V, Hornberger L, Zhang J, Han X (1999). Comparison of Chinese and caucasian families caregiving to children with cancer at home: part I. J Pediatr Nurs.

[41] Yao R, He C, Xiao P (2023). 'Food and medicine continuum' in the East and West: old tradition and current regulation. Chin Herb Med.

[42] Supreme Court of Korea (2004). Murder (acknowledged crime: aiding and abetting murder), murder. Case Law Information.

[43] Supreme Court of Korea (2009). Removal of meaningless life-sustaining treatment devices, etc. Case Law Information.

[44] Nolin T, Walther S (2021). The relationship between life-sustaining treatment limitation and organ donation in Swedish intensive care: a nationwide register study. Acta Anaesthesiol Scand.

[45] Mark NM, Rayner SG, Lee NJ, Curtis JR (2015). Global variability in withholding and withdrawal of life-sustaining treatment in the intensive care unit: a systematic review. Intensive Care Med.

[46] Kim CJ, Hong KS, Cho S, Park J (2024). Comparison of factors influencing the decision to withdraw life-sustaining treatment in intensive care unit patients after implementation of the life-sustaining treatment act in Korea. Acute Crit Care.

[47] Kim YJ, Kim SH (2023). Advance care planning in South Korea. Z Evid Fortbild Qual Gesundhwes.

[48] Lee YJ, Kim S, Yoo SH, Kim A, Lin C, Martina D, Mori M, Suh SY, Fhea, Sup, Sup (2024). Advance care planning in palliative care in Asia: barriers and implications. J Hosp Palliat Care.

[49] Salazar MM, Khera N, Chino F, Johnston E (2024). Financial hardship for patients with cancer and caregivers at end of life in the USA: narrative review. BMJ Support Palliat Care.

[50] Collado L, Brownell I (2019). The crippling financial toxicity of cancer in the United States. Cancer Biol Ther.

[51] Klaiman T, Steckel J, Hearn C, Diana A, Ferrell WJ, Emanuel EJ, Navathe AS, Parikh RB (2024). Hospice administrators' and providers' perspectives on providing upstream palliative care: facilitators, barriers, and policy prescriptions. J Palliat Med.

[52] Tang ST, Mccorkle R (2003). Determinants of congruence between the preferred and actual place of death for terminally ill cancer patients. J Palliat Care.

[53] Sheridan PE, LeBrett WG, Triplett DP, Roeland EJ, Bruggeman AR, Yeung HN, Murphy JD (2021). Cost savings associated with palliative care among older adults with advanced cancer. Am J Hosp Palliat Care.

[54] Luta X, Ottino B, Hall P, Bowden J, Wee B, Droney J, Riley J, Marti J (2021). Evidence on the economic value of end-of-life and palliative care interventions: a narrative review of reviews. BMC Palliat Care.

[55] Kim Y, Han E, Lee J, Kang H (2022). Cost-effectiveness analysis of home-based hospice-palliative care for terminal cancer patients. J Hosp Palliat Care.

[56] Kozlov E, Phongtankuel V, Prigerson H, Adelman R, Shalev A, Czaja S, Dignam R, Baughn R, Reid MC (2019). Prevalence, severity, and correlates of symptoms of anxiety and depression at the very end of life. J Pain Symptom Manage.

[57] Götze H, Brähler E, Gansera L, Schnabel A, Gottschalk-Fleischer A, Köhler N (2018). Anxiety, depression and quality of life in family caregivers of palliative cancer patients during home care and after the patient's death. Eur J Cancer Care (Engl).

[58] Vernon E, Hughes MC, Kowalczyk M (2022). Measuring effectiveness in community-based palliative care programs: a systematic review. Soc Sci Med.

[59] Highet G, Crawford D, Murray SA, Boyd K (2014). Development and evaluation of the supportive and palliative care indicators Tool (SPICT): a mixed-methods study. BMJ Support Palliat Care.

[60] Im H, Lee I, Kim S, Lee JS, Kim J, Moon JY, Park BK, Lee KH, Lee MA, Han S, Hong Y, Kim H, Cheon J, Koh S (2023). Experience and perspectives of end-of-life care discussion and physician orders for life-sustaining treatment of Korea (POLST-K): a cross-sectional study. BMC Med Ethics.

[61] Han SK, Eo Y (2022). Patients' dying process from the point of view of family and hospice team: a qualitative exploration of family member and hospice team experiences with hospice in Korea. Omega (Westport).

[62] Lee J, Kim J, Kim T, Kim C (2019). Communication about death and confidence levels concerning death-related issues among Koreans. Korean J Fam Pract.

[63] Ernecoff NC, Hanson LC, Fox AL, Daaleman TP, Kistler CE (2020). Palliative care in a community-based serious-illness care program. J Palliat Med.

[64] Bouazizi M, Ohtsuki T (2019). Multi-class sentiment analysis on twitter: classification performance and challenges. Big Data Min. Anal.

